# Mapping the intersection of nanotechnology and SARS-CoV-2/COVID-19: A bibliometric analysis

**DOI:** 10.1016/j.imj.2022.06.005

**Published:** 2022-06-26

**Authors:** Xuejuan Zhang, Mengqin Guo, Zhengwei Huang, Ying Huang, Chuanbin Wu, Xin Pan

**Affiliations:** a College of Pharmacy, Jinan University, Guangzhou 510632, China; b School of Pharmaceutical Sciences, Sun Yat-sen University, Guangzhou 510006, China

**Keywords:** Bibiometric analysis, Nano technology, SARS-CoV-2, COVID-19, Web of Science

## Abstract

**Background:**

Coronavirus disease 2019 (COVID-19), caused by infection with severe acute respiratory syndrome coronavirus 2 (SARS-CoV-2), has imposed great medical and economic burdens on human society, and nanotechnology is a promising technique for managing the ongoing COVID-19 pandemic. To drive further studies on anti-COVID-19 nanotechnology, this paper provides an analysis, from a bibliometric perspective, of the intersection of nanotechnology and SARS-CoV-2/COVID-19.

**Methods:**

We analyzed the 2585 publications on nanotechnology and SARS-CoV-2/COVID-19 included in the Web of Science Core Collection from January 2019 to March 2022 to determine the bibliometric landscape. The basic bibliometric characteristics are summarized in this article.

**Results:**

Our bibliometric analysis revealed that the intersection between nanotechnology and SARS-CoV-2/COVID-19 is a cutting-edge field in the science community and that the related studies were multidisciplinary in nature. Studies on the structural basis of SARS-CoV-2, SARS-CoV-2 detection assays, and mRNA vaccines against COVID-19 provided the development foundation for this field.

**Conclusions:**

The current research focuses are the development of nanomaterial-based vaccines and SARS-CoV-2 detection methods, and the design of nanomedicines carrying SARS-CoV-2 inhibitors is a relatively burgeoning frontier. In summary, this bibliometric analysis of the intersection of nanotechnology and SARS-CoV-2/COVID-19 highlights the current research focuses of this field to inspire future studies on anti-COVID-19 nanotechnologies.

## Graphical abstract

**Figure:**
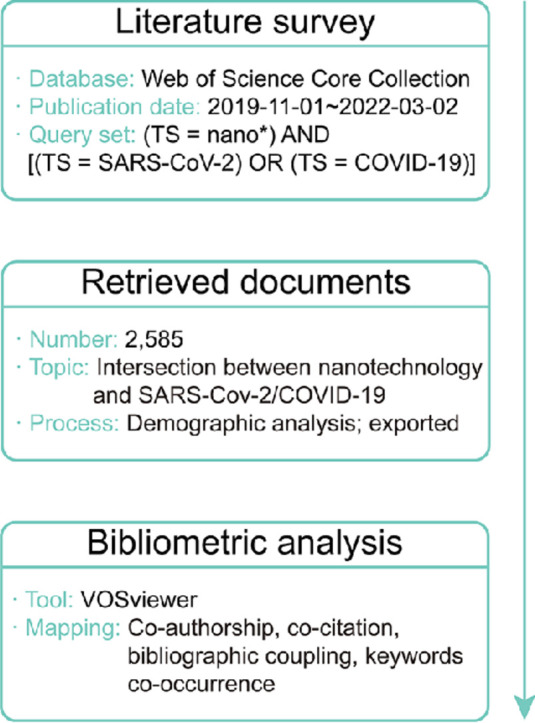

## 1 Introduction

Coronavirus disease 2019 (COVID-19) is an acute respiratory distress syndrome caused by a new type of coronavirus, severe acute respiratory syndrome coronavirus 2 (SARS-CoV-2) [1], which severely damages the lungs and also impacts the other major organs, including the heart, liver, and kidneys [2]. The infectivity of SARS-CoV-2 is quite high; the original strain has a net reproductive rate (*R*_0_) of 2.5, while that of the recent variants is nearly 7.0 [3]. Since the outbreak of COVID-19 began in December 2019, the pandemic has affected more than 200 countries/regions worldwide, causing almost 300 million cases and over 5 million deaths (data from Johns Hopkins University COVID Resource Center, January 2022 [4]). This pandemic has imposed great medical and economic burdens on the entire human society, and global efforts must be made to combat it.

Nanotechnology is a promising technique to use against COVID-19 and can be applied in the prevention, diagnosis, and treatment of this disease. First, nanometer-sized vaccines can be developed for the prevention of COVID-19 [5]. Second, nanosensors can be used for the rapid detection of SARS-CoV-2 [6]. Third, therapeutic drugs can be incorporated into nanocarriers to treat COVID-19 [7]. The products of nanotechnology hold promise for helping to surmount the COVID-19 pandemic.

Importantly, sophisticated and systemic laboratory investigation is always a prerequisite for the commercialization and clinical translation of nanotechnology [8]. Further studies on anti-COVID-19 nanotechnology need to be conducted to accumulate additional supportive evidence. To guide this work, it is advisable to summarize the current state-of-art technology, mine the research hot spots, and determine useful future directions for the field. Bibliometric analysis is an effective tool for fulfilling this aim [9]. At present, a few important bibliometric analyses regarding COVID-19 have been published [10], [11], [12], [13], [14], [15], but this is a dearth of specialized ones on the intersection of nanotechnology and COVID-19 (more information can be found in Supplemental Table S1).

Here, the intersection of nanotechnology and SARS-CoV-2/COVID-19 is reviewed from a bibliometric perspective. After document retrieval, the bibliometric properties, including the demographics, co-authorship, co-citation, bibliographic coupling, and keyword co-occurrence, of the literature were characterized. On the basis of the bibliometric analysis results, some future trends in this field are proposed. Because this bibliometric analysis reflects the research focuses of the field, the authors anticipate that it will inspire future studies regarding anti-COVID-19 nanotechnologies.

## 2 Methodology

A literature survey of the studies in the Web of Science Core Collection on both nanotechnology and SARS-CoV-2/COVID-19 was performed at 6 p.m., March 10, 2022 (Beijing Time). This database was used to ensure that the papers with highest impact were included [16]. The query set was “(TS = nano*) AND [(TS = SARS-CoV-2) OR (TS = COVID-19)]”, where “TS” indicates “topic”. Considering the time at which the original COVID-19 outbreak began [17], the publication date range was set as from November 1, 2019 to March 1, 2022.

The retrieved documents and the citation report generated from them were analyzed to describe the demographics [18]. The full record and cited references were exported as plain text files and visualized by VOSviewer software (Leiden University, Leiden, Netherlands) [19]. The applied mapping threshold was as follows. Co-authorship, authors: 5; co-authorship, organizations, 20; co-authorship, countries/regions: 50; bibliographic coupling: 100; co-citation: 100; keyword co-occurrence: 50. The counting method was full counting [20].

**Scheme 1:**
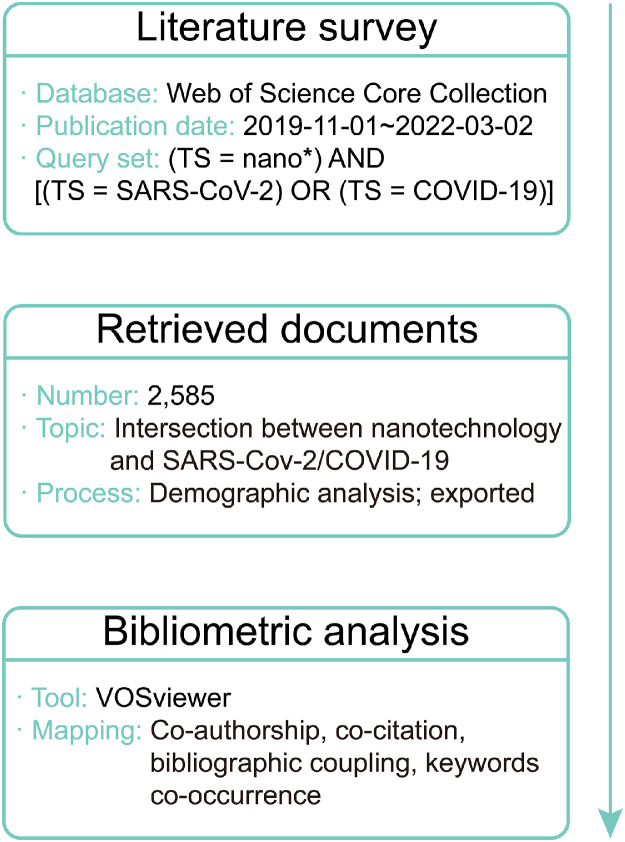
Flowchart scheme of this bibliometric review.

A flowchart scheme of the workflow for our bibliometric analysis is shown in Scheme 1.

## 3 Demographics

**Fig. 1:**
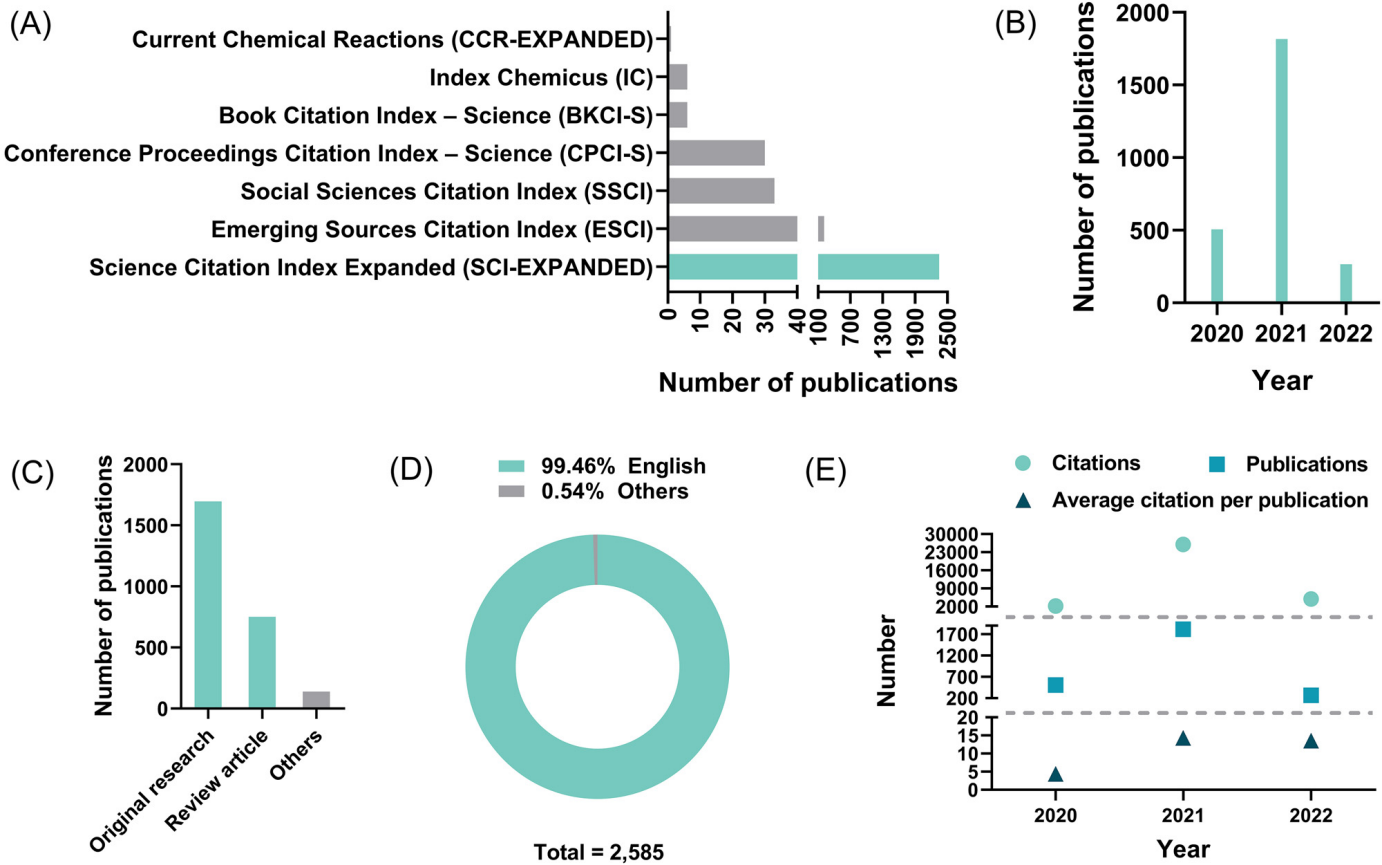
Demographics of the 2585 documents. (A) Indexed database; (B) Publication year; (C) Document type; (D) Language; (E) Citation performance by year.

The literature survey returned 2585 documents of interest. Among them, 470 were published in journals with a subscription model, and 2115 were published with open access. The large proportion of articles with open access facilitated correspondence regarding these publications and the propagation of their ideas [21]. According to the data shown in Fig. 1A, most of the documents of interest were indexed in the Science Citation Index Expanded database (2347, 90.79%), and a minor subset (238, 9.21%) of them was indexed in other Web of Science databases. The distribution of publications across publication years is shown in Fig. 1B. None of these documents was published in 2019 (Fig. 1C), and the investigation of nanotechnology for use in COVID-19 intervention began in 2020. A 3.59-fold increase in publications was demonstrated in the year 2021 compared with the year 2020, and a further increase is expected for 2022 and subsequent years. A majority (1696, 65.61%) and a considerable (750, 29.01%) portion of the documents were original research and review articles, respectively, and other document types, such as editorials, letters, and news accounted for 5.38% of the documents of interest. In terms of publication language, 99.46% of the documents were composed in English, which is favorable for international communication [22] (Fig. 1D). The citation performance of the documents is depicted in Fig. 1E, and, interestingly, the citation frequency was in parallel with the publication distribution across years (Fig. 1B). The number of times cited, cited publications, and average citations per cited publication increased by 11.61, 3.59, and 3.26 times, respectively, in 2021 compared with 2020. More citations will be observed in the following years.

More demographical data are provided in Supplemental Tables S2–S6. The top-10 research areas are listed in Supplemental Table S2. The documents were found to be associated mainly with material, pharmaceutical, microbiological, and immunological science. Supplemental Table S3 summarizes the top-10 journals in which the documents were published; they were in the fields of nanotechnology, analytical chemistry, drug delivery, and vaccinology. According to the data shown in Supplemental Table S4, the United States, China, and India were the top-3 contributing countries with over 300 documents each, whereas other countries/regions contributed less than 200 documents each. Not counting the United States, China, and India, 17 countries/regions (e.g., Iran, England, Italy) contributed at least 50 documents. There were 36 organizations that contributed at least 20 documents, and most of them were universities around the world (Supplemental Table S5). The top-10 funding agencies are shown in Supplemental Table S6. The investigators in this field are recommended to seek further financial support from these funding agencies.

The demographics results suggest that the intersection between nanotechnology and SARS-CoV-2/COVID-19 is a cutting-edge field in the science community and has provoked international concern and discussion. Furthermore, the related studies were found to be multidisciplinary in nature, arousing the interest of journals in different areas.

## 4 Co-authorship

The co-authorship behaviors for the 2585 documents were analyzed to determine the state of collaboration in this field. Specifically, we generated a co-authorship network among countries/regions, organizations, and authors by using VOSviewer software.

**Fig. 2:**
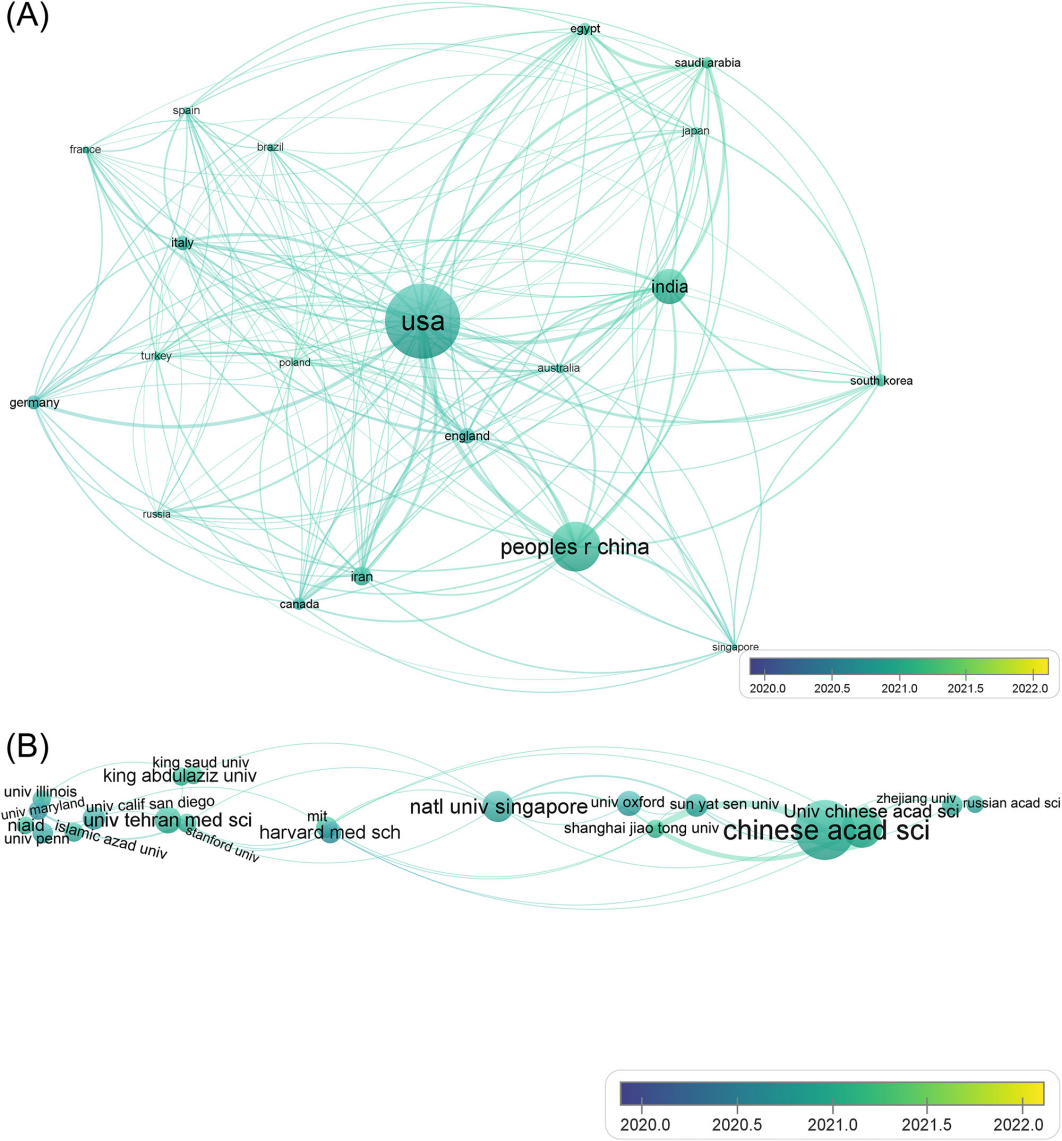
Co-authorship of the 2585 documents. (A) Countries/regions; (B) Organizations.

A visualization of the co-authorship of countries/regions is shown in Fig. 2A. Countries/regions in North America (e.g., United States), Asia (e.g., China), Oceania (e.g., Australia), Europe (e.g., Germany), South America (e.g., Brazil), and Africa (e.g., Egypt) that contributed at least 50 publications were involved. Overall, the resulting network was dense, demonstrating an active cooperation status. The average publication year for all countries/regions was 2021, suggesting a state of emerging collaborative relationships. The top-3 countries (United States, China, and India) are deposited like a triangle in Fig. 2A, indicating that they are serving as the cooperative centers. They cooperated with all other 19 countries/regions (link = 19). As seen from the link strength values, the United States established its strongest collaborations with China (link strength = 65), India (link strength = 45), and Germany (link strength = 37), China established its strongest collaborations with the United States (link strength = 65), England (link strength = 25), and Australia (link strength = 20), and India established its strongest collaborations with the United States (link strength = 45), Saudi Arabia (link strength = 30), and China (link strength = 18). The United States and China were the largest bilateral cooperators for each other, and the trilateral collaboration among the United States, China, and India was quite tight. Additionally, many countries/regions, including England, Australia, Canada, Turkey, Italy, and Iran, built cooperative tiles with all other 19 countries that were active collaborators in this field. The co-authorship network of countries/regions reflected the modes of North–North, North–South, and South–South cooperation [23], indicating that this topic demanded a global collaboration.

Fig. 2B depicts the profile of co-authorship of organizations (threshold: 20 publications). Worldwide distribution was demonstrated by the presence of organizations in the United States (e.g., Massachusetts Institute of Technology, abbreviated as MIT), China (e.g., Chinese Academy of Sciences), Singapore (e.g., National University of Singapore), and Iran (e.g., Tehran University of Medical Sciences). Similar to the data shown in Fig. 2A, the average publication year for all organizations was 2021. However, unlike the dense network shown in Fig. 2A, the distribution of items in Fig. 2B has a spindle-like shape. Intriguingly, the cooperative tiles amongst organizations were not as intense as those amongst countries/regions. As shown in Fig. 2B, National University of Singapore established the most cooperative tiles with other organizations (link = 8), followed by Chinese Academy of Science (link = 7), and MIT (link = 7). However, the collaborative relationship intensity of National University of Singapore and MIT was not strong. The strongest collaborative relationship was observed between Chinese Academy of Science and University of Chinese Academy of Sciences (link strength = 47). Shanghai Jiao Tong University established a moderate collaborative relationship with Chinese Academy of Science and University of Chinese Academy of Sciences, with link strengths of 7 and 5, respectively. The link strengths of other organization pairs were each lower than 5. Hence, the most active interinstitution cooperation occurred in Chinese organizations, but that in other countries/regions could not be neglected.

**Fig. 3:**
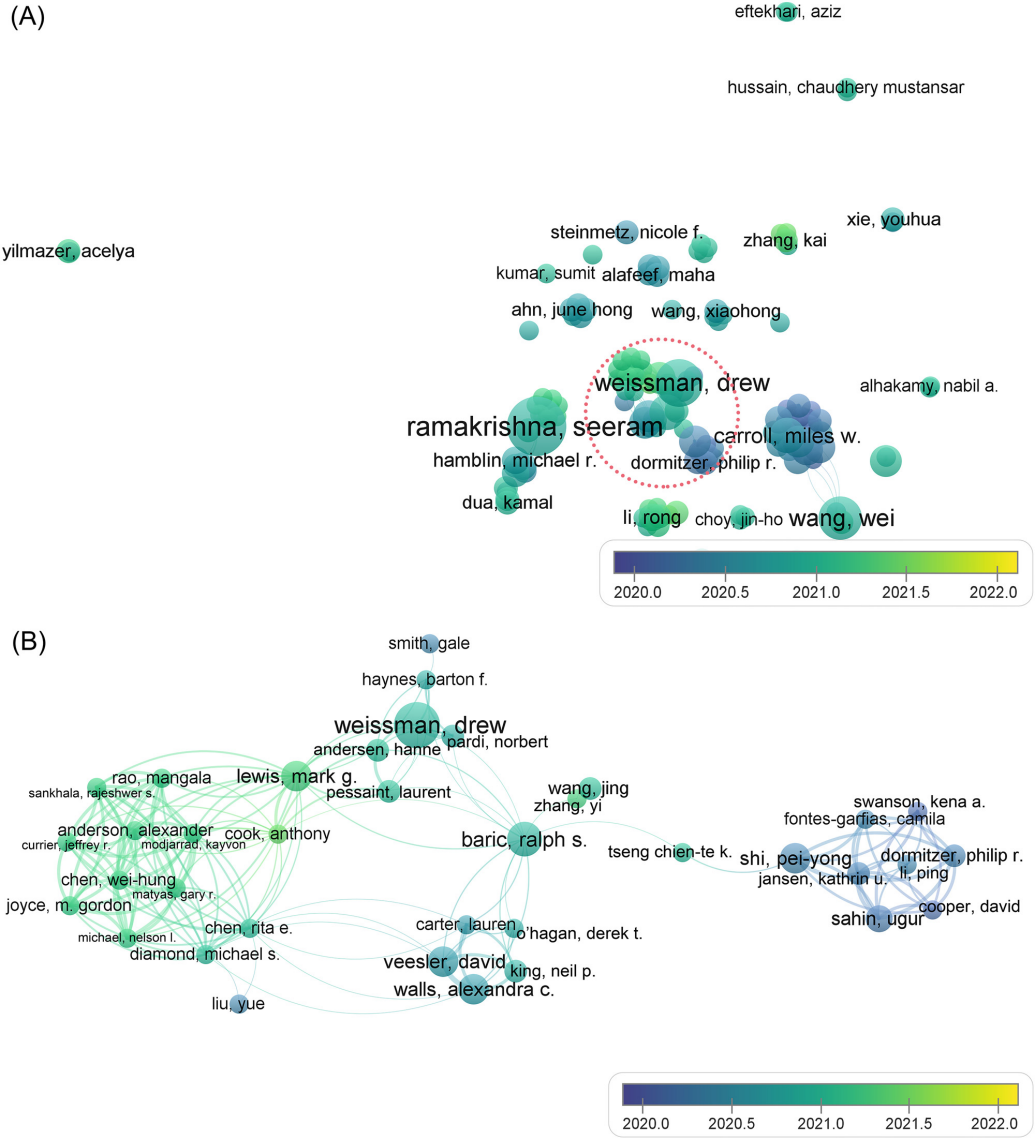
Co-authorship of the 2585 documents, in terms of researchers. (A) Full data set; (B) Magnification of the partial network of the red oval region in (A).

Subsequently, an analysis of the co-authorship of authors was conducted, and the results are displayed in Supplemental Figure S1 and Fig. 3. The authors who contributed at least 5 publications were categorized into 28 clusters in Supplemental Figure S1, indicating that there were at least 28 groups of independent investigators in this field. Herein, “independent” meant that no cooperation tiles had been built among these groups. Fig. 3A shows that the average publication time varied between February 2020 and March 2021, and the fluctuation was slightly greater than that shown in Fig. 2. Under the scattered distribution pattern, the cluster of Drew Weissman et al. (marked in the red oval) stood out as a central one. For a further interpretation of these authors’ co-authorship behavior, the detailed partial network of this cluster is magnified in Fig. 3B. Besides Drew Weissman (primary affiliation: University of Pennsylvania), Mark G. Lewis (primary affiliation: Bioqual Inc), Ralph S. Baric (primary affiliation: University of North Carolina at Chapel Hill), David Veesler (primary affiliation: University of Washington), and Pei-yong Shi (primary affiliation: University of Texas Medical Branch) were also pivots of the cooperation network. This analysis revealed that researchers from the United States participated in collaboration quite frequently. It might be advisable to seek cooperation with these researchers in the near future [24] because they were recently active in this field, as inferred from the average publication time.

According to the results of the co-authorship analysis, international cooperation in this field was a common phenomenon, and the United States, China, and India actively engaged in collaboration. Regarding organizations, Chinese organizations were the most active cooperators. Regarding investigators, those from the United States were the main collaboration pivots. Cooperation would boost the development of relevant studies [25] and bring new insights for COVID-19 therapy.

## 5 Co-citation and bibliographic coupling

To connect the bibliographic information from the 2585 documents and explore the development of this field, co-citation and bibliographic coupling analyses were performed [26]. Co-citation was defined as 2 referenced documents being cited by a retrieved document, and bibliographic coupling was defined as a referenced document that was cited by 2 retrieved documents [27].

**Fig. 4:**
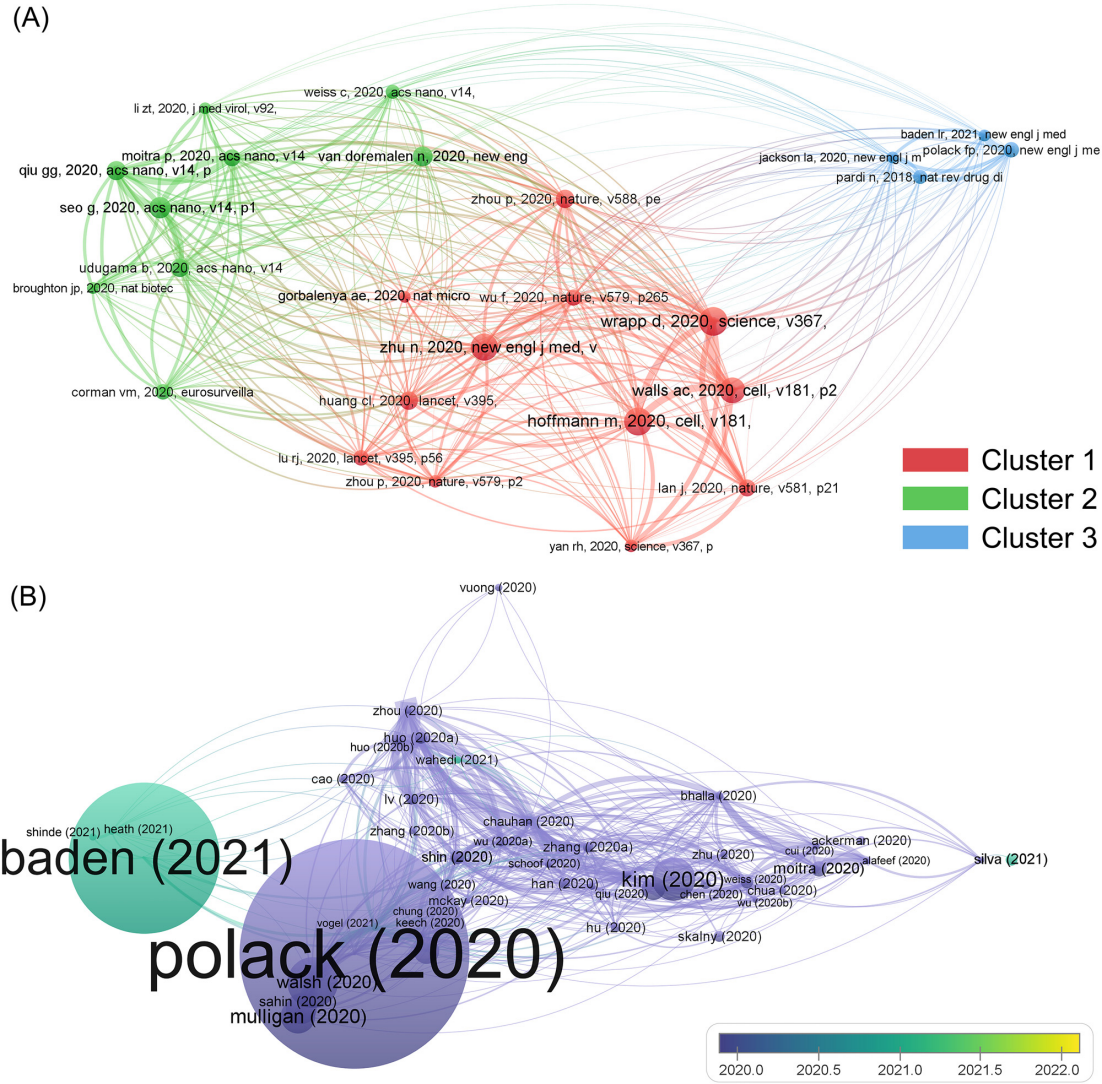
Bibliographic information of the 2585 documents. (A) Clustered co-citation visualization; (B) Time-overlay bibliographic coupling visualization.

The results of the co-citation analysis, with a threshold of 100, are presented in Fig. 4A. This analysis classified the documents into 3 clusters. Clusters 1, 2, and 3 were mainly about the structural basis of SARS-CoV-2 [28], [29], [30], detection assays for SARS-CoV-2 [31], [32], [33], and mRNA vaccines against COVID-19 [34], [35], [36], respectively. Noticeably, in this analysis, papers not related to the intersection of nanotechnology and SARS-CoV-2/COVID-19 were involved, such as (N. Pardi, 2018, *Nature Reviews Drug Discovery*) [37]. Research in the above three areas laid the referenced foundation for this field, and it may offer inspiration regarding the applied methodologies and mechanisms [22].

With regards to bibliographic coupling, the clustered results can be found in Supplemental Figure S2. As in the co-citation analysis, the threshold value was set as 100. Herein, 5 clusters were obtained, and generally documents within the same cluster shared a similar research intention or paradigm [38]. The representative documents for the 5 groups were: Kim (2020) [39], discussing the SARS-CoV-2 transcriptome; Lv (2020) [40], introducing the structural basis for the neutralization antibodies against SARS-CoV and SARS-CoV-2; Polack (2020) [36], evaluating the BNT162b2 mRNA vaccine for SARS-CoV-2; Han (2020) [41], designing SARS-CoV-2 inhibitors by using sequences from angiotensin-converting enzyme 2; and Baden (2021) [34], evaluating the mRNA-1273 vaccine for SARS-CoV-2. Most representative documents were about treatment strategies for SARS-CoV-2 [34], especially mRNA vaccines; the prevailing research intention or paradigm could be speculated from their topics.

The bibliographic network was then redrawn with a time-overlay pattern, displayed in Fig. 4B. A majority of papers were published in 2020, and only a small proportion was documented in 2021. Baden (2021), mentioned above, was a representative document published in 2021. The other 4 papers documented in 2021 are: Heath (2021) [42], evaluation of the NVX-CoV2373 vaccine for SARS-CoV-2; Shinde (2021), evaluation of the NVX-CoV2373 vaccine for the B.1.351 variant of SARS-CoV-2 [43]; Wahedi (2021) [44], computer-aided design of stilbene derivatives for COVID-19 treatment; and Silva (2021) [45], scenario regarding the plastic pollution during the COVID-19 pandemic. The evaluation of vaccines against the SARS-CoV-2 variants, discovery of chemotherapy drugs for treating COVID-19, and the environmental change associated with the pandemic emerged as new topics in this field. It is believed that more attention will likely be paid to these aspects in the future.

In summary, studies on the structural basis of SARS-CoV-2, SARS-CoV-2 detection assays, and mRNA vaccines against COVID-19 formed the development basis for this field, and the prevailing research mode was attempting to establish therapeutic strategies for COVID-19. By analyzing the bibliographic information, the current publishing paradigm for the intersection of nanotechnology and SARS-CoV-2/COVID-19 was determined.

## 6 Keyword co-occurrence

**Fig. 5:**
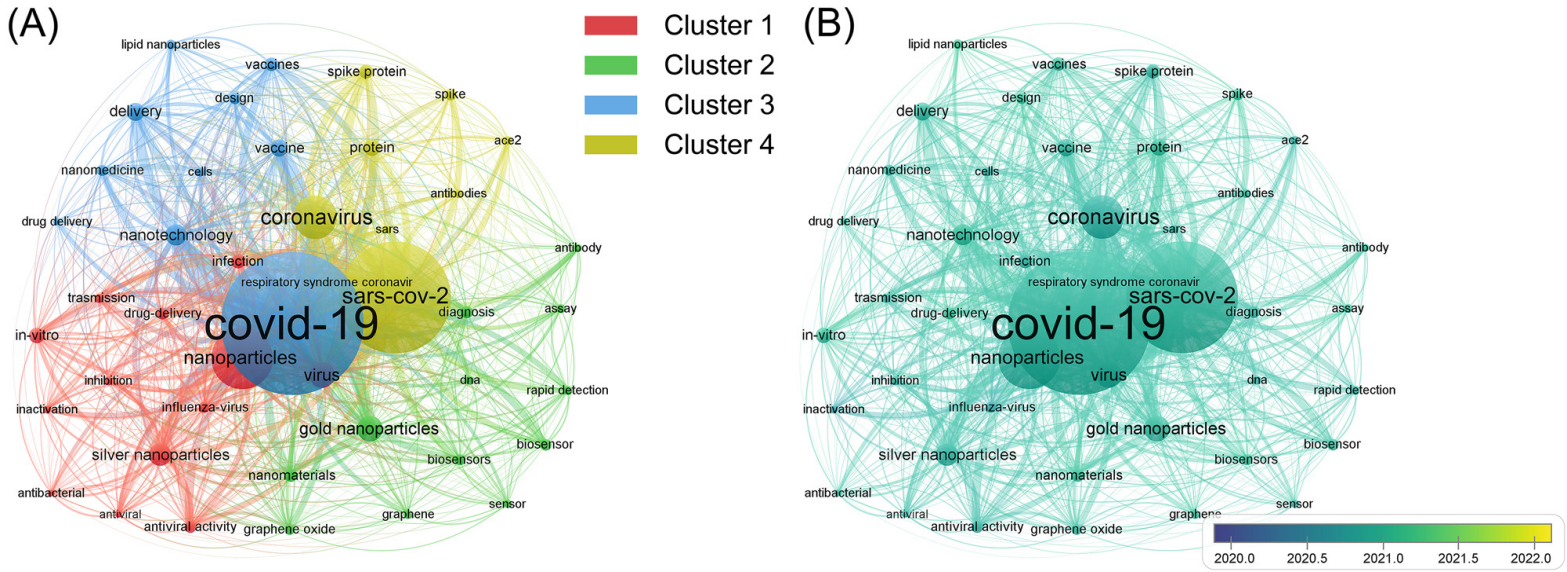
Keyword co-occurrence of the 2585 documents. (A) Clustered visualization; (B) Time-overlay visualization.

Lastly, all the keywords used by the 2585 documents were sorted to investigate the research hot spots in this field on a microscopic level [46]. The keyword co-occurrence in these documents is presented in Fig. 5. The keywords with the highest frequency (≥50 occurrences) were divided into four clusters (Fig. 5A). The clustered keywords in Clusters 1–4 reflect the 4 research hot spots in this field. For Cluster 1 (nanoparticulate systems for delivering drugs with anti-SARS-CoV-2 activity), typical keywords included: “nanoparticles,” “inhibition,” “drug-delivery,” and “antiviral activity”. For Cluster 2, (nanotechnology for rapid and quantitative SARS-CoV-2 detection), typical keywords included: “gold nanoparticles,” “diagnosis,” “rapid detection,” and “biosensor”. For Cluster 3 (nanovaccines against SARS-CoV-2 delivered by lipid nanoparticles), typical keywords included: “COVID-19,” “nanotechnology,” “vaccine,” and “lipid nanoparticles”. For Cluster 4 (screening antibodies for SARS-CoV-2 neutralization on the basis of viral structure), typical keywords included: “SARS-CoV-2,” “coronavirus,” “spike protein,” and “antibodies”. The closed-loop network suggested that the 4 hot spots could have overlap [47]. Furthermore, according to the data shown in Fig. 5B, the distribution of the average publication time for all keywords was highly homogeneous (∼2021). Therefore, it was inferred that the above four hot spots are still under discussion in the science community, and more related studies can be expected.

The keyword co-occurrence results indicate that the current research hot spots are: nanoparticulate systems for delivering drugs with anti-SARS-CoV-2 activity, nanotechnology for rapid and quantitative SARS-CoV-2 detection, nanovaccines against SARS-CoV-2 delivered by lipid nanoparticles, and screening antibodies for SARS-CoV-2 neutralization on the basis of viral structure. By combining these results with those of the co-citation and bibliographic coupling analyses, it was found that the development of nanomaterial-based vaccines and detection methods for SARS-CoV-2 has been the current research focus and that the design of nanomedicines carrying SARS-CoV-2 inhibitors is a relatively burgeoning frontier.

## 7 Future trends

From the results of this bibliometric analysis, the future trends of this field were predicted. The following discussion is anticipated to help interested scientists identify fruitful new research avenues.

Studies on COVID-19 vaccine and detection assay development will likely continue to be reported, and the generation of new vaccines and detection assays, particularly those for new and severe SARS-CoV-2 variants (e.g., Delta [48] and Omicron [49]), will probably be emphasized. For example, the development of nasal-delivered nanovaccines [50] has gained much attention, and frugal diagnostic approaches, like RNA-extraction-free nanotechnology, for SARS-CoV-2 detection [51] also inspired considerable research interest.

Several drugs have been recommended for use in the clinical therapy of SARS-CoV-2, including α-interferon [52], ribavirin [53], arbidol [54], and chloroquine phosphate [55]. Additionally, traditional Chinese medicines, such as *Maxing Shigan* decoction, *Qingfei Paidu* decoction, and *Dayuan* decoction, were confirmed as effective remedies for COVID-19 [56]. Transforming these drugs into nanomedicines, especially inhalable ones, could further increase their therapeutic effects and mitigate their adverse reactions [57]. Thus, studies on nanocarriers for the delivery of anti-SARS-CoV-2 drugs might also be a productive area of future research.

## 8 Limitations of this work

The main flaw of the presented study is that only one database, the Web of Science Core Collection, was used for document retrieval. Although the quality of documents could be guaranteed by using only this respected database, some interesting contributions that are not indexed in this database were consequently omitted [58]. As a result, some research topics in this field were probably ignored. In our ongoing study, the list of targeted databases will be expanded to include Scopus [59], PubMed [60], and Google Scholar [61] to incorporate as many relevant publications of interests as possible.

## 9 Conclusion

In this work, the intersection of nanotechnology and SARS-CoV-2/COVID-19 was analyzed from a bibliometric perspective. First, the basic bibliometric characteristics were evaluated. The results show that the intersection between nanotechnology and SARS-CoV-2/COVID-19 has been a cutting-edge field in the science community and studies on this topic have provoked international concern and discussion. Co-authorship and international cooperation was a common phenomenon in this field, and the United States, China, and India actively took part in collaborations. Second, the results of co-citation and bibliographic coupling analyses show that studies on the structural basis of SARS-CoV-2, SARS-CoV-2 detection assays, and mRNA vaccines against COVID-19 served as the development basis for this field and that the prevailing research mode has been to establish therapeutic strategies for COVID-19. Third, hotspot mining was achieved through the analysis of keyword co-occurrence. This revealed that nanoparticulate systems delivering drugs with anti-SARS-CoV-2 activity, nanotechnology for rapid and quantitative SARS-CoV-2 detection, nanovaccines against SARS-CoV-2 delivered by lipid nanoparticles, and screening antibodies for SARS-CoV-2 neutralization on the basis of viral structure have been the current research hot spots. Overall, this work provides a bibliometric picture of the interplay between nanotechnology and SARS-CoV-2/COVID-19 studies.

## Author contribution

X. Zhang: Manuscript composing and artwork preparation. M. Guo: Literature survey, bibliometric analysis and software operation. Z. Huang: Data double-checking and format adjustment. Y. Huang: Supervision and proof-reading. C. Wu: Conceptualization and manuscript polishing. X. Pan: Supervision and conceptualization.

## Data available statement

No data, models, or code were generated or used during the study.

## Declaration of competing interest

The authors declare that they have no known competing financial interests or personal relationships that could have appeared to influence the work reported in this paper.

## Funding

This study was supported by the 10.13039/501100001809National Natural Science Foundation of China (82104070 and 82104072).

## Ethic statement

This research reported has adhered to the Jinan University ethical guidelines.

## Informed consent

Not applicable.

## Appendix Supplementary materials

Image, application 1
