# Supplementary material for: Mapping the intersection of nanotechnology and SARS-CoV-2/COVID-19: A bibliometric analysis
Source: Infect Med (Beijing). 2022 Jun 26;1(2):103–12. doi: 10.1016/j.imj.2022.06.005 (PMC9233748; doi:10.1016/j.imj.2022.06.005)
Supplement: Supplementary file 1 [file mmc1.docx]

**Mapping the Intersection of Nanotechnology and SARS-CoV-2/COVID-19: A Bibliometric Review**

Xuejuan **Zhang**^1,#^, Mengqin **Guo**^1,#^, Zhengwei **Huang**^1,*^, Ying **Huang**^1^, Chuanbin **Wu**^1^, Xin **Pan**^2^

*(1 College of Pharmacy, Jinan University, Guangzhou 510632, P. R. China*

*2 School of Pharmaceutical Sciences, Sun Yat-sen University, Guangzhou 510006, P. R. China)*

# Equal contributing authors: Xuejuan Zhang and Mengqin Guo.

* Corresponding author: Zhengwei Huang Ph.D.. Tel: 020-39943117.

**——Supplementary material——**

**Tab. S1** Differences between this work and previous studies.

| Article | Database | Timespan | Software | Focus |
| --- | --- | --- | --- | --- |
| **This work** | **Web of Science Core Collection** | **Nov. 1st, 2019 ~ Mar. 1st, 2022** | **VOSviewer** | **Interplay between nanotechnology and COVID-19** |
| [10]* | Web of Science Core Collection | 2019 to May. 20^th^, 2020 | VOSviewer | A comprehensive study |
| [11] | Web of Science, Scopus and Google Scholar | ~May. 29^th^, 2020 | - | The top cited papers |
| [12] | Web of Science Core Collection | 2019 to Jul. 2020 | VOS viewer, Biblioshiny and BibExcel | A comprehensive study |
| [13] | Science Citation Index Expanded database, bioRxiv, medRxiv, Preprints and SSRN | ~Oct. 14^th^, 2020 | - | A comprehensive study |
| [14] | Embase, Scopus, hinese databases Chinese Biomedical Database, CNKI, VIP and Wanfang | ~Mar. 1^st^, 2020 | VOSviewer and CiteSpace | Comparison of research between English and Chinese studies |
| [15] | Web of Science, Scopus, and PubMed | ~Sep. 6^th^, 2020 | R | Dental scientific contributions |

* referred to reference number in the main text:

[10] Y.T. Yu, Y.J. Li, Z.H. Zhang, Z.C. Gu, H. Zhong, Q.F. Zha, L.Y. Yang, C. Zhu, E.Z. Chen, A bibliometric analysis using VOSviewer of publications on COVID-19, Ann Transl Med, 8 (2020).

[11] H. ElHawary, A. Salimi, N. Diab, L. Smith, Bibliometric Analysis of Early COVID-19 Research: The Top 50 Cited Papers, Infect Dis (Auckl), 13 (2020) 1178633720962935.

[12] R.K. Farooq, S.U. Rehman, M. Ashiq, N. Siddique, S. Ahmad, Bibliometric analysis of coronavirus disease (COVID-19) literature published in Web of Science 2019-2020, J Family Community Med, 28 (2021) 1-7.

[13] P. Wang, D. Tian, Bibliometric analysis of global scientific research on COVID-19, J Biosaf Biosecur, 3 (2021) 4-9.

[14] J.C. Fan, Y. Gao, N. Zhao, R.J. Dai, H.L. Zhang, X.Y. Feng, G.X. Shi, J.H. Tian, C. Chen, B.D. Hambly, S.S. Bao, Bibliometric Analysis on COVID-19: A Comparison of Research Between English and Chinese Studies, Front Public Health, 8 (2020).

[15] J. Jacimovic, A. Jakovljevic, V. Nagendrababu, H.F. Duncan, P.M.H. Dummer, A bibliometric analysis of the dental scientific literature on COVID-19, Clin Oral Invest, 25 (2021) 6171-6183.

It was shown that although a couple of bibliometric studies have been published regarding COVID-19, they were mainly comprehensive studies or specific studies irrelevant to nanotechnology. In other words, the presented work was different the previous studies in terms of the research focus. Hence, it is meaningful to perform a bibliometric study upon the intersection between COVID-19 and nanotechnology.

**Tab. S2** Top-10 research areas of the 2,585 documents.

| No. | Research area | Count | Percentage |
| --- | --- | --- | --- |
| 1 | Chemistry | 841 | 32.53 |
| 2 | Science & Technology, Other Topics | 739 | 28.59 |
| 3 | Materials Science | 644 | 24.91 |
| 4 | Pharmacology & Pharmacy | 322 | 12.46 |
| 5 | Physics | 272 | 10.52 |
| 6 | Biochemistry & Molecular Biology | 252 | 9.75 |
| 7 | Engineering | 196 | 7.58 |
| 8 | Biotechnology & Applied Microbiology | 156 | 6.04 |
| 9 | Research & Experimental Medicine | 154 | 5.96 |
| 10 | Immunology | 108 | 4.18 |

**Tab. S3** Top-10 publication titles of the 2,585 documents.

| No. | Publication title | Count | Percentage |
| --- | --- | --- | --- |
| 1 | ACS Nano | 57 | 2.21 |
| 2 | Biosensors & Bioelectronics | 56 | 2.17 |
| 3 | Scientific Reports | 36 | 1.39 |
| 4 | Nanomaterials | 35 | 1.35 |
| 5 | Vaccines | 31 | 1.20 |
| 6 | ACS Applied Materials & Interfaces | 29 | 1.12 |
| 7 | Pharmaceutics | 27 | 1.04 |
| 8 | Analytical Chemistry | 26 | 1.01 |
| 8 | International Journal of Molecular Sciences | 26 | 1.01 |
| 10 | Journal of Controlled Release | 25 | 0.97 |
| 10 | Nano Today | 25 | 0.97 |
| 10 | Nature Communications | 25 | 0.97 |

**Tab. S4** Top contributing countries/regions with at least 50 publications of the 2,585 documents.

| No. | Country/region | Count | Percentage |
| --- | --- | --- | --- |
| 1 | USA | 713 | 27.58 |
| 2 | China | 476 | 18.41 |
| 3 | India | 340 | 13.15 |
| 4 | Iran | 181 | 7.00 |
| 5 | England | 151 | 5.84 |
| 6 | Italy | 144 | 5.57 |
| 7 | Germany | 132 | 5.11 |
| 8 | Canada | 123 | 4.76 |
| 8 | South Korea | 123 | 4.76 |
| 10 | Saudi Arabia | 116 | 4.49 |
| 11 | Egypt | 110 | 4.26 |
| 12 | Australia | 95 | 3.68 |
| 13 | Turkey | 80 | 3.10 |
| 14 | Brazil | 79 | 3.06 |
| 15 | Spain | 77 | 2.98 |
| 16 | Japan | 74 | 2.86 |
| 17 | France | 71 | 2.75 |
| 18 | Singapore | 59 | 2.28 |
| 19 | Russia | 55 | 2.13 |
| 20 | Poland | 50 | 1.93 |

**Tab. S5** Top contributing organizations with at least 20 publications of the 2,585 documents.

| No. | Organization | Count | Percentage |
| --- | --- | --- | --- |
| 1 | Egyptian Knowledge Bank | 107 | 4.14 |
| 2 | Chinese Academy of Sciences | 89 | 3.44 |
| 3 | University of California System | 82 | 3.17 |
| 4 | University of Chinese Academy of Sciences | 49 | 1.90 |
| 5 | University of Texas System | 48 | 1.86 |
| 6 | Indian Institute of Technology System | 45 | 1.74 |
| 7 | Centre National de la Recherche Scientifique | 42 | 1.63 |
| 8 | Harvard University | 36 | 1.39 |
| 9 | National University of Singapore | 35 | 1.35 |
| 10 | Tehran University of Medical Sciences | 33 | 1.28 |
| 11 | Council of Scientific Industrial Research | 32 | 1.24 |
| 11 | National Institutes of Health (NIH) | 32 | 1.24 |
| 13 | Massachusetts Institute of Technology (MIT) | 29 | 1.12 |
| 14 | Consiglio Nazionale Delle Ricerche | 28 | 1.08 |
| 14 | University of Oxford | 28 | 1.08 |
| 16 | Harvard Medical School | 26 | 1.01 |
| 16 | Sun Yat-sen University | 26 | 1.01 |
| 16 | University of California, San Diego | 26 | 1.01 |
| 16 | University System of Maryland | 26 | 1.01 |
| 20 | Islamic Azad University | 25 | 0.97 |
| 20 | State University System of Florida | 25 | 0.97 |
| 20 | University of London | 25 | 0.97 |
| 23 | Russian Academy of Sciences | 24 | 0.93 |
| 23 | University of Pennsylvania | 24 | 0.93 |
| 25 | Stanford University | 23 | 0.89 |
| 25 | University of North Carolina | 23 | 0.89 |
| 25 | Zhejiang University | 23 | 0.89 |
| 28 | King Abdulaziz University | 22 | 0.85 |
| 28 | King Saud University | 22 | 0.85 |
| 30 | Pennsylvania Commonwealth System of Higher Education | 21 | 0.81 |
| 30 | Shanghai Jiao Tong University | 21 | 0.81 |
| 30 | University of Illinois System | 21 | 0.81 |
| 30 | University System of Georgia | 21 | 0.81 |
| 34 | Chinese Academy of Medical Sciences, Peking Union Medical College | 20 | 0.77 |
| 34 | NIH National Institute of Allergy & Infectious Diseases (NIAID) | 20 | 0.77 |
| 34 | University of California, Los Angeles | 20 | 0.77 |

**Tab. S6** Top-10 funding agencies of the 2,585 documents.

| No. | Funding agency | Count | Percentage |
| --- | --- | --- | --- |
| 1 | United States Department of Health Human Services | 256 | 9.90 |
| 2 | National Institutes of Health (NIH) | 253 | 9.79 |
| 3 | National Natural Science Foundation of China | 246 | 9.52 |
| 4 | European Commission | 113 | 4.37 |
| 5 | National Science Foundation | 102 | 3.95 |
| 6 | Natural Sciences and Engineering Research Council of Canada | 53 | 2.05 |
| 6 | NIH National Institute of Allergy & Infectious Diseases (NIAID) | 53 | 2.05 |
| 6 | UK Research & Innovation | 53 | 2.05 |
| 9 | Department of Science & Technology, India | 45 | 1.74 |
| 10 | Conselho Nacional de Desenvolvimento Cientifico e Tecnologico | 38 | 1.47 |


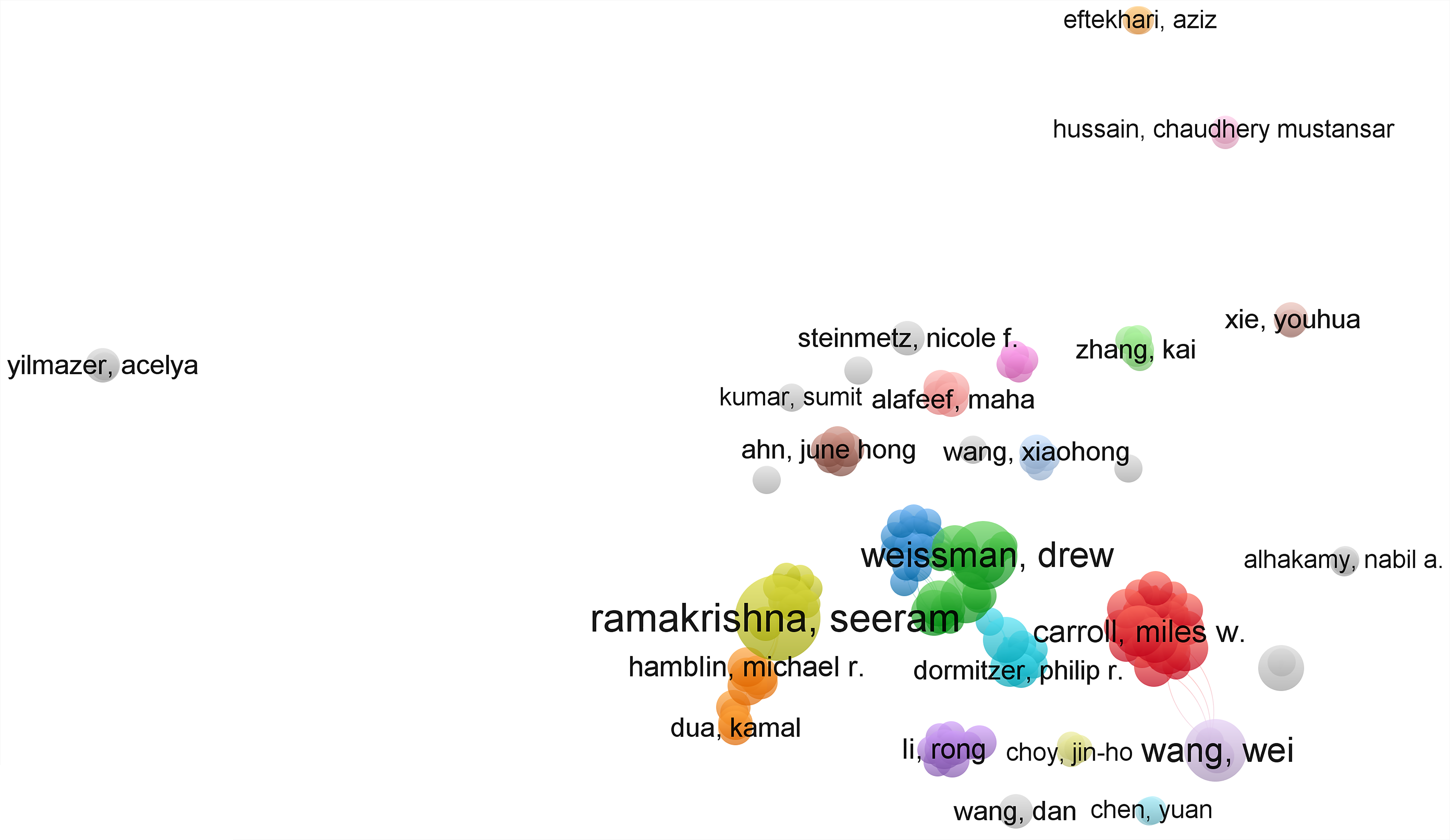


**Fig. S1** Co-authorship network of authors with at least 5 publications


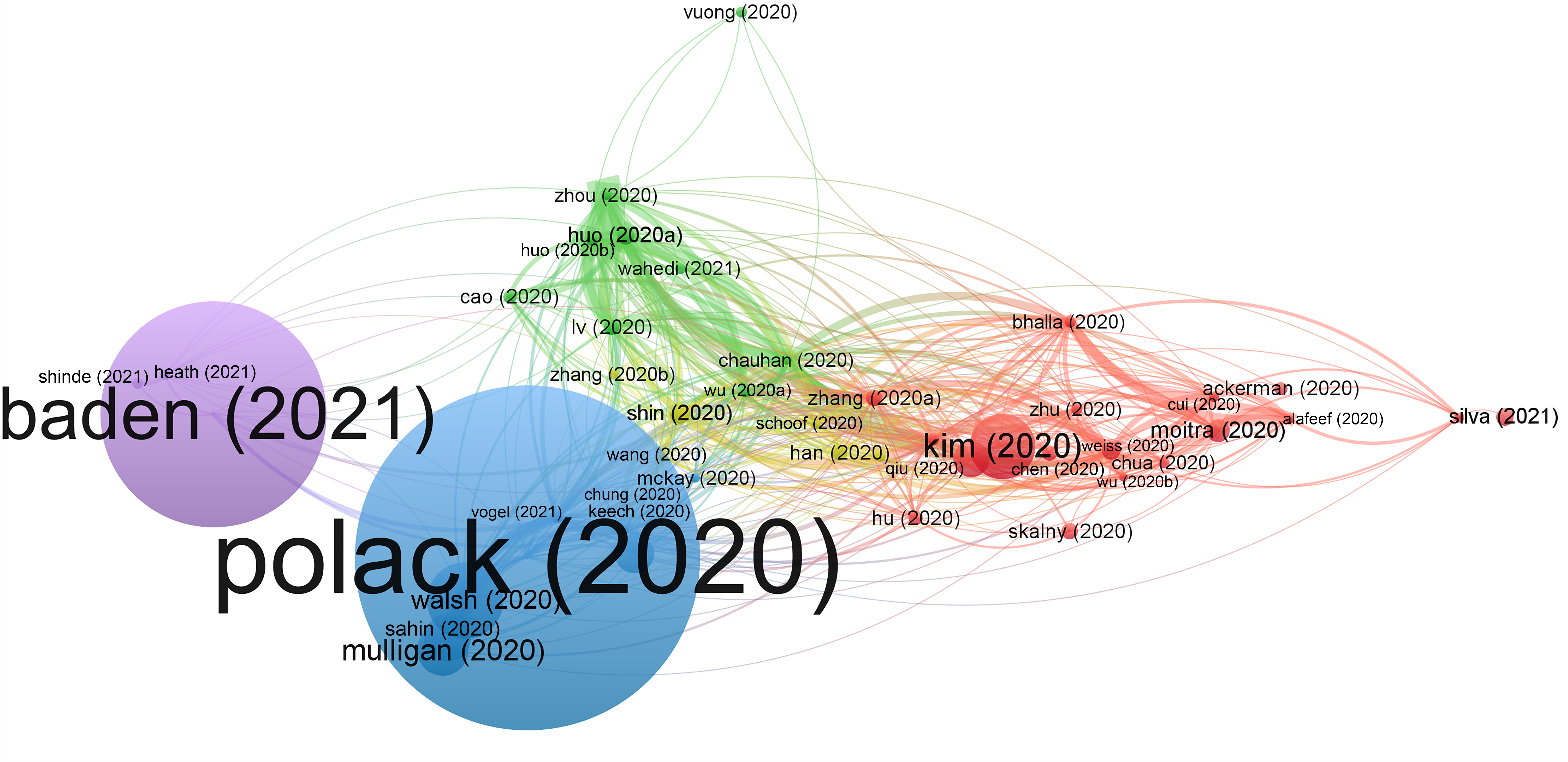


**Fig. S2** Bibliographic coupling network over the threshold of 100.
